# Theoretical and electrochemical performance of Quinazoline Schiff-base hybrid as levelling agents for C-steel electropolishing in acidic medium

**DOI:** 10.1038/s41598-025-25001-8

**Published:** 2025-11-20

**Authors:** Amira H. E. Moustafa, Hanaa H. Abdel-Rahman, Seleim M. Seleim, Asmaa M. Embaby, Alaa Z. Omar

**Affiliations:** Chemistry Department, Faculty of Science, Alexandria University, P.O. 426, Ibrahemia, Alexandria, 21321 Egypt

**Keywords:** Electropolishing (EP), Schiff-base, Levelers, Adsorption, Surface topography, Molecular modeling, Corrosion resistance, Chemistry, Materials science

## Abstract

This study leverages an innovative knowledge of electrolytic polishing (EP) mechanisms by introducing a newly synthesized Quinazolin Schiff-base hybrid (*N*-(arylidene)-2-(4-oxoquinazoline-3(4H)-yl) acetohydrazide **(QA)**) as a levelling agent for C-steel surfaces in 8M H_3_PO_4_. Several spectroscopic methods, including FT-IR, ^1^H NMR, and ^13^C NMR, were employed to verify the chemical structures of **QA** derivatives. EP with **QA** derivatives, particularly *N'*-(3-Methylbenzylidene)-2-(4-oxoquinazolin-3(4H)-yl) acetohydrazide (***m-CH3-QA***), reduced surface roughness by **83.24%**, smoothing defects. **QA** adsorption on C-steel followed a physical adsorption mechanism, supported by kinetic and thermodynamic analyses. Scanning electron microscopy (**SEM**), water contact angle (**WCA)** measurements, and atomic force microscopy (**AFM**) confirmed improved hydrophilicity/hydrophobicity and a protective corrosion-resistant layer. Energy-dispersive X-ray (**EDX**) spectroscopy revealed changes in surface composition due to **QA** adsorption. Computational analyses, including Chemical Reactivity Descriptors, Molecular Electrostatic Potential (**MEP**), Fukui indices, and Local Dual Descriptors, identified strong interactions between **QA** molecules and *Fe* ions at specific atomic sites. These findings demonstrate how **QA** derivatives enhance surface quality through adsorption, resulting in fewer imperfections than untreated surfaces and a promising approach for improving C-steel surfaces. In addition to theoretical calculations, atomic absorption spectroscopy (**AAS**) was employed as an alternative to gravimetric techniques. The results from both approaches align well with each other and with those obtained through the electrochemical method.

**Supplementary Information:**

The online version contains supplementary material available at 10.1038/s41598-025-25001-8.

## Introduction

Due to its widespread availability, high efficiency, cost-effectiveness, and diverse mechanical properties, C-steel is extensively utilized across various industrial sectors. Broadly, C-steel is considered an inexpensive alloy that offers better corrosion resistance compared to others. As it is used in multiple fields, such as power plants, petroleum industries, and pharmaceutical mixers, it tends to corrode when exposed to acidic media. C-steel loses its integrity due to the progressive deterioration resulting from corrosion when interacting with aggressive environments. Corrosion has harmful effects that extend to natural and historic monuments as well as infrastructure^1,2^. The cost of conservation and substitution of corroded materials alone is predicted to account for between 3 and 4% of the national growth product (GNP)^3^.

Hence, a groundbreaking technique in the field of corrosion research is the corrosion of C-steel in an acidic solution, particularly in relation to the use of organic inhibitors. As a result, surface modification is substantial to cope with aggressive conditions in different fields. In recent decades, numerous research studies have focused on surface treatment techniques interacting with surrounding biological environments. Electropolishing (EP) has been recommended as a feasible method for enhancing embedded corrosion resistance in physiological settings^4^.

A while ago, researchers affirmed that EP is much more influential for the C-steel smoothing process rather than mechanical polishing, which develops cold contortion and remnant stresses, where EP dissolves more nonmetallic involvements, such as manganese sulphides and chromium to a large extent^5^. The produced crystal structure of the smoothened C-steel surface after EP ensures its sterility for usage in crucial industries such as food processing and pharmaceutical fields, since metal components that directly contact food cannot contaminate it or alter its flavor or appearance. These benefits of EP encourage the use of this technique for completing surgical tools^6^.

During electropolishing, burrs and rough surfaces erode faster due to higher current densities. That comes from the difference in dissolution rates of the estimated metal or alloy surface, which is dependent on the current division or mass-transportation provisions. This is termed anodic levelling^7^. Organic additives in the EP bath include (i) brighteners, (ii) carriers, and (iii) levelers. Levelers improve the surface finish on carbon steel, act as grain refiners, widen the electropolishing window, and enhance the solubility of wetting agents^8,9^.

Most levelers are nitrogen-containing organic compounds that stabilize *Fe* ions during EP, preventing decomposition and improving current density and hole-filling rates by inhibiting Fe dissolution^10,11^. However, existing levelers are often costly and environmentally harmful. There is a need to develop affordable, eco-friendly, and easily synthesized alternatives to facilitate commercialization^12–14^.

Bountiful contexts have validated the efficacy of heteroatom compounds in inhibiting EP. This affinity is for possessing a π-bond system or aromatic rings with functional groups with different structures and electron densities. Nevertheless, *N*, *O*, and *S* are heteroatoms with accessible electron pairs that are prominently important in inhibition due to their unique interaction with the c-steel surface. Combining these two characteristics results in enhanced EP inhibition^15^.

Due to their remarkable anti-corrosion properties, eco-friendliness, and application in various fields as catalysts and optical properties, Quinazoline Schiff-base hybrid has gained more and more attention as a levelling agent or as a film-forming corrosion inhibitor across different metals and environments, underscoring their pivotal role in developing novel inhibitors^16–18^. Quinazolinones and their Schiff’s bases constitute an important class of heterocyclic compounds. They occupy an essential position in medicinal and pesticide chemistry, presenting a wide range of bioactivities. These are frequently encountered heterocycles in medical chemistry, with broad applications, having antitumor properties^19,20^, antimicrobial^21–23^, anticonvulsant^24^, antidepressant and antihistaminic^25^, anti-inflammatory^26,27^, antimalarial^28,29^, diuretic and hypertensive agents^30^, and dihydrofolate reductase (DHFR) inhibition^31^.

Based on the low cost of starting materials and the ease of synthesis, a new strategy for preparing highly pure, low-toxicity, and eco-friendly corrosion inhibitors using an innovative Quinazolin Schiff-base hybrid compound with high interfacial compatibility was developed in the present work. These compounds contain some molecular features that are regarded as a leveler agent for the first time and are designed to reduce internal surface roughness. On the other hand, to elucidate and correlate the different molecular structures of Quinazolin derivatives QA with their leveler efficiency, we apply the DFT theory here to understand the surface interaction required to achieve the beneficial electrochemical characteristics.

## Experimental approach

### Recognition of leveler agents (QA)

Fourier-transform infrared (FTIR) spectra were acquired on a Bruker Tensor 37 spectrometer (Central Laboratory, Faculty of Science, Alexandria University) to analyze the **QA** structure. ^1^HNMR and ^13^CNMR spectra were reported on a Jeol-500 MHz, ECA-500 II spectrometer (field strength of 11.74 T, Japan, in DMSO-d6; Faculty of Science, Mansoura University). The Micro Analytical Unit at Cairo University’s Faculty of Science performed elemental analyses.

### Leveler agents’ synthesis

#### Synthesis of *N*'-(arylidene)-2-(4-oxoquinazoline-3(4H)-yl)acetohydrazide (QA)

**Scheme 1:**
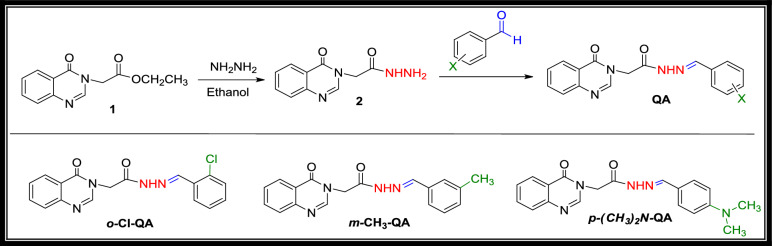
Schematic pathway for synthesis of *N*'-(arylidene)-2-(4-oxoquinazoline-3(4*H*)-yl)acetohydrazide **QA**.

A mixture of 2-(4-oxoquinazolin-3(4*H*)-yl)acetohydrazide **2** (2.18 g, 0.01 mol) and substituted benzaldehyde (0.01 mol) (Namely, 2-chlorobenzaldehyde, 3-methylbenzaldehyde, and 4-(dimethylamino)benzaldehyde) in ethanol (30 mL) was refluxed for 5–8 h. The reaction was monitored with Thin Layer Chromatography (TLC). The products (**QA**) were filtered and washed with ethanol, and no further purification was required (Scheme 1).

### Leveler agents’ characterization

#### *N*'-(2-Chlorobenzylidene)-2-(4-oxoquinazolin-3(4*H*)-yl)acetohydrazide *o-Cl*-QA

Colorless crystals, 88% yield; m.p. 260 °C. IR (KBr): 3239 (N–H), 3059 (sp^2^ = C–H), 1685 (C=O), 1607 (C=O), and 1560 (C=N) cm^−1^ Fig. (S1). ^1^H NMR (400 MHz: DMSO-*d6*): 11.99 (Br.s. 1H, NH, D_2_O exchangeable), 8.45 (s, 1H, N=CH), 8.36 (s, 1H, H-2), 8.17 (d, *J* = 7.8 Hz, 1H, H-5), 8.05 (d,* J* = 7.1 Hz, 1H, H-6^/^), 7.88 (t, *J* = 7.6 Hz, 1H, H-7), 7.73 (d, *J* = 8.1, 1H, H-8), 7.60 (t, *J* = 7.4 Hz, 1H, H-6), 7.54 (d, *J* = 7.2 Hz, 1H, H-3^/^), 7.45 (m, 2H, H-4^/^ and H-5^/^) and 5.24 (s, 2H, N-CH_2_) ppm Fig. (S2-S4). ^13^C APT NMR (101 MHz: DMSO-d6): δ 168.96 (C = O, hydrazide), 160.88 (C = O, lactam), 148.36 (C), 141.08 (CH), 135.19 (CH), 133.54 (C), 132.12 (CH), 131.48 (C), 130.46 (CH), 128.16 (CH), 127.83 (CH), 127.63 (CH), 127.50 (CH), 127.42 (CH), 126.52 (CH), 121.74 (C) and 47.56 (CH_2_) ppm Fig. (S5). C_17_H_13_ClN_4_O_2_ requires: C: 59.92; H: 3.85; N: 16.44% found: C: 60.13; H: 4.07; N: 16.28%.

#### *N*'-(3-Methylbenzylidene)-2-(4-oxoquinazolin-3(4*H*)-yl)acetohydrazide *m-CH*_*3*_*-*QA

Colorless crystals, 91% yield; m.p. 260 °C. IR (KBr): 3282 (N–H), 3056 (sp^2^ = C–H), 2924 (sp^3^ -C-H), 1677 (C=O), 1612 (C=O), and 1551 (C=N) cm^−1^ Fig. (S6). ^1^H NMR (400 MHz: DMSO-d6): 11.73 (Br.s. 1H, NH, D_2_O exchangeable), 8.35 (s, 1H, N=CH), 8.16 (d, *J* = 7.8 Hz, 1H, H-5), 8.03 (s, 1H, H-2), 7.87 (t,* J* = 7.6 Hz, 1H, H-7), 7.73 (d, *J* = 8.2 Hz, 1H, H-8), 7.54 (m, 3H, H-6, H-2^/^ and H-6^/^), 7.35 (t, *J* = 7.3 Hz, 1H, H-5^/^), 7.25 (d, *J* = 7.3 Hz, 1H, H-4^/^), 5.22 (s, 2H, N-CH_2_) and 2.34 (2, 3H, CH_3_) ppm Figs. (S7, S8). ^13^C APT NMR (101 MHz: DMSO-d6): δ 168.73 (C=O, hydrazide), 160.93 (C=O, lactam), 149.00 (CH), 148.31 (C), 145.29 (CH), 138.71 (C), 135.19 (CH), 134.17 (C), 131.43 (CH), 129.30 (CH), 127.83 (CH), 127.69 (CH), 127.57 (CH), 126.50 (CH), 124.80 (CH), 121.67 (C), 47.59 (CH_2_) and 21.30 (CH_3_) ppm Fig. (S9, S10). C_18_H_16_N_4_O_2_ requires: C: 67.49; H: 5.03; N: 17.49% found: C: 67.24; H: 5.31; N: 17.61%.

#### *N*'-(4-(Dimethylamino)benzylidene)-2-(4-oxoquinazolin-3(4*H*)-yl)acetohydrazide *p-(CH*_*3*_*)*_*2*_*N*-QA

Yellow crystals, 87% yield; m.p. 260 °C. IR (KBr): 3294 (N–H), 3066 (sp^2^ = C–H), 2912 (sp^3^ -C-H), 1698 (C=O), 1643 (C=O), and 1565 (C=N) cm^-1^ Fig. (S11). ^1^H NMR (400 MHz: DMSO-d6): 11.52 (Br.s. 1H, NH, D_2_O exchangeable), 8.37 (s, 1H, N=CH), 8.17 (d, *J* = 8.0 Hz, 1H, H-5), 7.93 (s, 1H, H-2), 7.88 (t, *J* = 7.5 Hz, 1H, H-7), 7.73 (d, *J* = 8.2 Hz, 1H, H-8), 7.54 (m, 3H, H-6, H-2^/^ and H-6^/^), 6.76 (d, *J* = 8.3 Hz, 2H, H-3^/^ and H-5^/^), 5.19 (s, 2H, N-CH_2_) and 2.98 (s, 6H, 2CH_3_) ppm Fig. (S12, S13). ^13^C APT NMR (101 MHz: DMSO-d6): δ 168.08 (C = O, hydrazide), 160.77 (C = O, lactam), 151.98 (C), 148.63 (CH), 148.53 (C), 145.67 (CH), 135.00 (CH), 129.01 (CH), 128.72 (CH), 127.63 (CH), 126.52 (CH), 121.87 (C), 121.61 (C), 112.27 (CH), 47.49 (CH_2_) and 40.22 (2CH_3_) ppm Figs. (S14, S15). C_19_H_19_N_5_O_2_ requires: C: 65.32; H: 5.48; N: 20.04% found: C: 65.19; H: 5.65; N: 19.89%.

### Elaboration of electropolishing electrolytes

Annular grade H_3_PO_4_ (85%W/W), supplied by BDH Chemicals Ltd., was diluted with double-distilled H_2_O to compose 8M H_3_PO_4_ as an acidic EP solution. The preselected organic compounds for EP retardation were N’–(arylidene)-2-(4 oxoquinazoline-3(4H)-yl) acetohydrazide **(QA)** derivatives**.** The **QA** concentrations varied between 0.33 × 10^−3^ and 3.43 × 10^−3^ mol/L. A relevant quantity of inhibitors was dissolved in 8 M H_3_PO_4_ and warmed to produce inhibition solutions, as illustrated in Table 1. The experiments were reliably triplicated to guarantee the measurements with less than 2% variation. The mentioned corrosion results are obtained by averaging the three measurements. All experiments were conducted with and without varying quantities of **QA**.

**Table 1:** Properties of synthesized **QA** compounds.

Inhibitor code	Name		Appearance		Structure	M.wt (g/mol)
**QA**	N'-(arylidene)-2- (4-oxoquinazoline-3(4H)-yl)					
***m*-CH**_**3**_**-QA**	N'-(3-Methylbenzylidene)-2- (4-oxoquinazolin-3(4H)yl)acetohydrazide		Colorless crystals		X = 3-CH_3_, M.wt = 320.52	
***o*-Cl-QA**	N'-(2-Chlorobenzylidene)-2- (4-oxoquinazolin-3(4H)-yl)acetohydrazide		Colorless crystals		X = 2-Cl, M.wt = 340.86	
***p*-(CH**_**3**_**)**_**2**_**N-QA**	N'-(4-(Dimethylamino)benzylidene)-2- (4-oxoquinazolin-3(4H)-yl)acetohydrazide		Yellow crystals		X = 4-(CH_3_)_2_N, M.wt = 349.43	

### Substrate elaboration

In our current research, the scoped-out C-steel has the following chemical composition: 0.285% C, 0.012% S, 0.221% Mn, 0.012% P, 0.13% Si, and balance Fe. Two electrodes, an anode and a cathode, are arranged in a diagonal configuration, measuring 10 cm in height, 5 cm in width, and 1 cm in breadth. Before each test, electrodes are manually polished using multiple kinds of sandpaper until they have a glossy surface, rinsed several times with deionized water, and decontaminated with acetone to remove undesirable film from the C-steel substrate. The epoxy adhesive was applied to cover the anode and cathode backs and surfaces, except for a C-steel specimen (4 × 5 cm^2^) used for Galvanostatic tests.

### Galvanostatic electrochemical assessment

The Galvanostatic approach was uncomplicated and dedicated, offering a viable standard for evaluating inhibitor effectiveness. The polarization experiments were conducted following previously described protocols^4,32^. Inhibition efficiency can be determined from polarization plots, which define the limiting current (EP rate) by gradually increasing the applied current and calculating the resulting stable-state voltage. The Inhibition efficiency (**%IE**) could be estimated from the following Equation:

1 $$\%IE=\frac{{I}_{l\left(B\right)}-{I}_{l(QA)}}{{I}_{l(B)}}\times 100$$

where ${I}_{l(B)}$ is the rate of EP in a **QA-**free solution**,** and ${I}_{l(QA)}$ is the rate of EP during the existence of **QA**.

### Surface spectroscopy/microscopy methods

- *Microstructure analysis:* The surface of all samples, measuring 1.5 cm × 1.5 cm, was extensively examined for morphological modification and chemical composition using a scanning electron microscope (SEM, JEOL JSM-5300) and an X-ray spectrometer (EDX), respectively. This phenomenon had been observed before, and subsequent involvement in 8M H3PO4 solution with and without varying concentrations of formulated **QA** at different temperatures.
- *UV–visible reflectance spectroscopy*: (Jasco V-570) was used to evaluate the reflection bands and surface illumination of C-steel specimens in the 200–800 nm range.
- *Wettability:* The concept of “surface wettability” is commonly measured using the apparent static water contact angle (**WCA**). This angle was described as the angle created where the liquid–vapor boundary of a droplet meets the solid–liquid interface. As a result, the adhesion of **QA** compounds affects surface properties, enabling a deeper study of hydrophilicity/hydrophobicity. Thus, contact angle analysis is necessary before and after EP. Measurements were taken using an **OCA15** goniometer (**DataPhysics Instruments, Filderstadt, Germany**), placing a 0.2 µL water droplet on the surface. The wettability angle was determined between the surface and the droplet’s tangent. Results were averaged from three measurements per sample, with dynamic changes tracked over time. Accuracy is ± 0.01.
- *Roughness exploration and estimating method:* An atomic force microscope (AFM) (thermos-microscope, auto probe CP-head, MLTC by Bruker, IP 2.1 software) was used to assess the average surface roughness factor (Ra) at two distinct locations in the central portion of a 25 × 25 μm^2^ region under various conditions. The measurements were conducted for three different areas in the samples. The average values are reported, and the standard deviations were included to reflect the precision and reproducibility of the measurements.SD values reflect < 5% relative erro.
- *Spectroscopic techniques*
  *Atomic absorption spectroscopy measurements (AAS):* The amount of iron ions *Fe*^2+^ released into solution due to corrosion and after Galvanostatic analysis in the absence and presence of the **QA**, was quantitatively analyzed using an atomic absorption spectrometer (ANALYTIK JENA CONTRAA 300). The experiment involved immersing C-Steel sheets in 8M H₃PO₄ as a corrosive medium, both with and without varying concentrations of QA derivatives at temperatures (298–313 K) for 10 minutes. The experiments were reliably triplicated with a variation of less than 2%.
- *Quantum chemical calculations*: using DFT carried out by the Gaussian 09W program package created by Frisch and collaborators, were used to perform all computational calculations on a personal computer. All computations were performed using “Becke’s three-parameter hybrid functional” employing the “LYP correlation functional B3LYP”, one of the most robust of the hybrid family, with a 6.31G (d,p) basis set^33,34^.

## Results and discussion

### QA synthesis and structure characterization

The new *N*'–(arylidene)-2-(4-oxoquinazolin-3(4*H*)-yl)acetohydrazide derivatives (**QA**) were synthesized as outlined in Scheme 1. The starting material, 2-(4-oxoquinazolin-3(4*H*)-yl)acetohydrazide **2**, was obtained by stirring ethyl 2-(4-oxoquinazolin-3(4*H*)-yl)acetate **1** with hydrazine hydrate in ethanol. The acetohydrazide derivative **2** was then condensed with variously substituted aromatic aldehydes (2-chlorobenzaldehyde, 3-methylbenzaldehyde, and 4-(dimethylamino)benzaldehyde) under reflux in ethanol to obtain the target acetohydrazides **QA** in a high yield.

The structures of quinazoline derivatives **QA** were elucidated using FT-IR, ^1^H NMR, ^13^C NMR spectroscopy, and elemental analysis. The FT-IR spectra of product **QA** displayed N–H stretching vibration in regions 3294–3239 cm^−1^ and weak absorption bands at 3066–3056 cm^−1^, which were attributed to the vibration of the aromatic C-H bond. Moreover, compounds **QA** showed strong absorption bands in the regions 1698–1677 and 1643–1607 cm^−1^ for the lactam and carbohydrazide carbonyl groups, respectively^35,36^. Furthermore, the medium absorption bands at 1612–1565 cm^−1^ were assigned to the vibration of the C=N group^37^. Additionally, compounds ***m-*CH**_**3**_***-QA*** and ***p*-(CH**_**3**_**)**_**2**_**N-QA** have absorption bands at 2924 and 2912 cm^−1^ ascribed to the stretching vibration of the aliphatic C-H bond.

Proton NMR (DMSO-*d*_6_) of compounds **QA** showed three singlet signals corresponding to the exchangeable NH (at δ 11.99, 11.73, and 11.52 ppm), azomethine CH=N (at δ 8.45, 8.35, and 8.37 ppm) and methylene protons (at δ 5.24, 5.22, and 5.19 ppm) for compounds ***o-*Cl*-QA, m-*CH**_**3**_***-QA*** and ***p*-(CH**_**3**_**)**_**2**_**N-QA**, respectively. Furthermore, the ^1^H NMR spectra of **QA** showed multiple signals in the range of 8.36–6.76 ppm associated with the aromatic rings’ protons derived from quinazolineyl and phenyl moieties. Additionally, the methyl substituents in the benzylidene moiety exhibited a singlet at 2.34 and 2.98 ppm in ^1^H NMR and at 21.30 and 40.22 ppm in ^13^C NMR spectra for ***m-*CH**_**3**_***-*QA** and ***p*-(CH**_**3**_**)**_**2**_**N-QA**, respectively.

### Determination of EP Action of QA

**Fig. 1:**
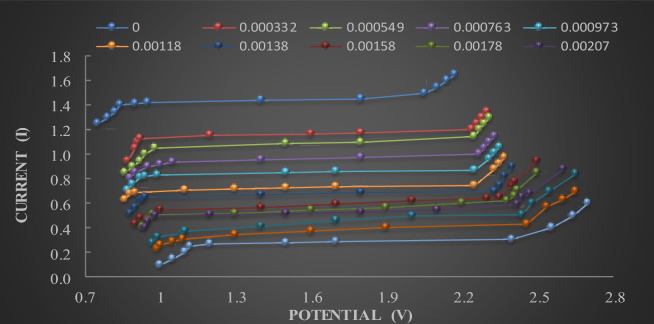
Current–potential curves for the electropolishing process of C-steel surfaces in the absence and presence of ***m*-CH**_**3**_**-QA** different concentrations at 298 K.

To provide a clue about EP kinetic reactions, the Galvanostatic technique is utilized to establish polarization procedures. For example, the polarization plots of C-steel before and after adding various concentrations of ***m*-CH**_**3**_**-QA** to 8 M H3PO4 are shown in Fig. 1. Examination of the polarization plots revealed that the plateau area represented the limiting current (I_L_) values at a specific temperature (T). Clearly, the values of I_L_, which are collected in Table 2, diminish as increasing **QA** derivative concentrations increase. This can be attributed to the large-scale mass transfer and the inferiority of double-layer fullness. The role of mass transfer is evident in altering the C-steel surface structure, leading to the levelling process, as protrusions on the C-steel surface diffuse more readily than indentations during EP. However, the amount of anodic dissolution propagates by mounting (T) from 298 to 313K, as exhibited in Table 2^38^.

**Table 2:** Magnitudes of ${I}_{l}$ and %IE for EP of C-steel in 8M H_3_PO_4_ and various concentrations of **QA** at temperatures varied from 298 to 313 K.

Levelling Agent	C*10^–3^ (mol/L)	298K		303K		308K		313K	
		${I}_{l}$(A)	%IE	${I}_{l}$(A)	%IE	${I}_{l}$(A)	%IE	${I}_{l}$(A)	%IE
***m*-CH**_**3**_**-QA**	Blank	1.85	0.00	2.2	0.00	2.37	0.00	2.50	0.00
	0.332	1.20	35.14	1.57	28.64	1.80	24.05	1.91	23.60
	0.549	1.15	37.84	1.42	35.45	1.75	26.16	1.86	25.60
	0.763	1.00	45.95	1.27	42.27	1.65	30.38	1.76	29.60
	0.973	0.87	52.97	1.15	47.73	1.56	34.18	1.66	33.60
	1.18	0.75	59.46	1.02	53.64	1.40	40.93	1.57	37.20
	1.38	0.70	62.16	0.93	57.73	1.36	42.62	1.46	41.60
	1.58	0.65	64.86	0.87	60.45	1.27	46.41	1.38	44.80
	1.78	0.62	66.49	0.75	65.91	1.20	49.37	1.32	47.20
	2.07	0.59	68.11	0.71	67.73	1.15	51.48	1.26	49.60
	2.54	0.50	72.97	0.67	69.54	1.08	54.43	1.23	50.80
	3.00	0.43	76.76	0.65	70.45	0.99	58.23	1.14	54.40
	3.43	0.31	**83.24**	0.55	**75.00**	0.70	**70.46**	1.05	**58.00**
***o*-Cl-QA**	Blank	1.85	0.00	2.20	0.00	2.37	0.00	2.50	0.00
	0.332	1.30	29.73	1.65	25.00	1.89	20.25	2.12	15.20
	0.549	1.25	32.43	1.45	34.09	1.86	21.52	2.01	19.60
	0.763	1.11	40.00	1.33	39.55	1.75	26.16	1.87	25.20
	0.973	0.93	49.73	1.22	44.55	1.58	33.33	1.75	30.00
	1.18	0.84	54.59	1.12	49.09	1.50	36.71	1.62	35.20
	1.38	0.76	58.92	0.98	55.45	1.45	38.82	1.58	36.80
	1.58	0.70	62.16	0.89	59.55	1.36	42.62	1.41	43.60
	1.78	0.66	64.32	0.82	62.73	1.24	47.68	1.35	46.00
	2.07	0.63	65.95	0.80	63.64	1.14	51.90	1.30	48.00
	2.54	0.57	69.19	0.75	65.91	1.10	53.59	1.28	48.80
	3.00	0.52	71.89	0.68	69.09	1.01	57.38	1.18	52.80
	3.43	0.43	**76.76**	0.58	**73.64**	0.92	**61.18**	1.11	**55.60**
***p*-(CH**_**3**_**)**_**2**_**N-QA**	Blank	1.85	0.00	2.20	0.00	2.37	0.00	2.50	0.00
	0.332	1.35	27.03	1.70	22.73	1.99	16.03	2.22	11.20
	0.549	1.31	29.19	1.50	31.82	1.94	18.14	2.05	18.00
	0.763	1.22	34.05	1.36	38.18	1.78	24.89	1.91	23.60
	0.973	1.07	42.16	1.29	41.36	1.63	31.22	1.76	29.60
	1.18	1.00	45.95	1.17	46.82	1.56	34.18	1.72	31.20
	1.38	0.90	51.35	1.05	52.27	1.48	37.55	1.68	32.80
	1.58	0.87	52.97	1.00	54.55	1.39	41.35	1.54	38.40
	1.78	0.85	54.05	0.92	58.18	1.28	45.99	1.50	40.00
	2.07	0.83	55.14	0.85	61.36	1.18	50.21	1.48	40.80
	2.54	0.81	56.22	0.83	62.27	1.16	51.05	1.43	42.80
	3.00	0.77	58.38	0.82	62.73	1.08	54.43	1.33	46.80
	3.43	0.72	**61.08**	0.78	**64.55**	1.04	**56.12**	1.25	**50.00**

**Fig. 2:**
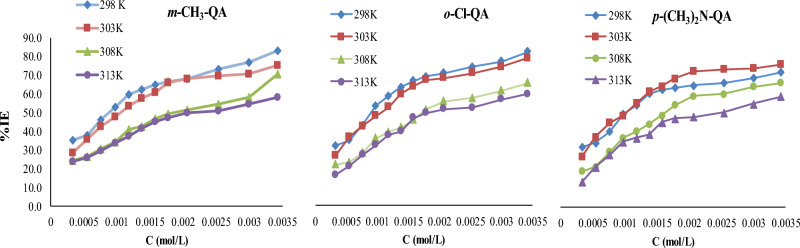
An analysis for %IE and concentration of **QA** derivatives at different temperatures ranges.

The tabulated data disclosed that higher concentrations of **QA** resulted in larger magnitudes of % IE at an analogous temperature. This referred to the extensive adsorption of the extreme **QA** concentrations on C-steel and their protective behavior efficiency. Furthermore, a decrease in %IE values with increasing temperature, at a similar **QA** concentration, would clarify that the growing temperature promotes the desorption of **QA** molecules from C-steel anode^39,40^ Fig. 2 and Table 2.

The efficiency of the retardation process can be ordered as follows: ***m-*CH**_**3**_ $>$ ***o-*Cl** > ***p-N, N***. This sequence is attributed to the molecule size, substituent groups, and adsorbed atoms from the functional group during the inhibition process^32^.

Additionally, two main features could control the effect of our compounds on the EP manner of C-steel in 8M H_3_PO_4,_ which are:

1. The stability of the metal complex, which is adsorbed on the anode during the process of EP retardation.
2. Solubility of the metal complex, which has a catalytic effect.

Taking into account that **QA** contains aromatic rings with functional groups as –CH_3_ and -*N, N,* and Cl atoms, the scope of the two complexes to be formed could exist. In advance, adsorption of the metal complex for the retardation effect has the leading role till the optimum concentration, where the retardation impact is extreme. After a while, the growing influence is suppressed by increasing the **QA** concentration^41,42^.

### Parameters for thermodynamic and activation

Activated parameters lead to more investigation of EP suppression to confirm the spontaneity of the C-steel’s EP process when exposed to different environmental conditions. The Arrhenius equation (Eq. 2) was employed to investigate the temperature dependence of the EP rate:

2 $$\text{ln}{I}_{l}=-\left(\frac{{E}_{a}}{RT}\right)+\text{ln}A$$

**Fig. 3:**
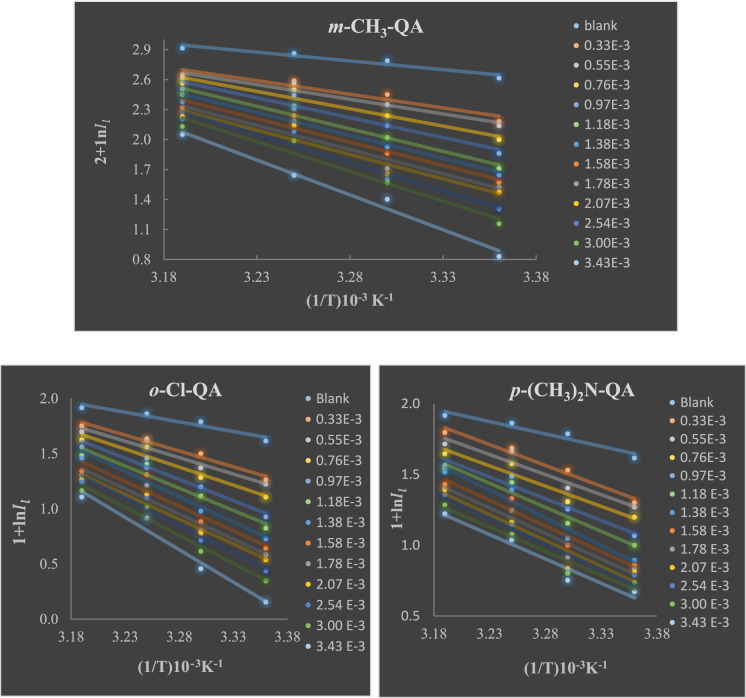
Arrhenius graphs for EP of C-steel specimen at various concentrations of **QA** derivatives.

Whereas the rate of EP is symbolized by ${I}_{l}$, *R* is the gas constant, *T* is the temperature, *A* refers to the pre-exponential factor that emulates the capacity of **QA** adsorption on C-steel, and ${E}_{a}$ is the activation energy for the EP reaction. *E*_*a*_ can be determined by plotting $\text{ln}{I}_{l}$ against *1/T,* as shown in Fig. 3.

According to the findings observed in Table 3, higher **QA** concentrations led to elevated *E*_*a*_​ values (e.g., 60.58 kJ/mol for ***m*-CH₃-QA** at 3.43 × 10⁻^3^ mol/L vs. 15.22 kJ/mol for the blank), demonstrating that **QA** adsorption creates an energy barrier, hindering mass transfer and anodic dissolution by occupying a more significant proportion of inhibitor molecules at the interface as the concentration increases. It is worth noting that the Arrhenius constant (*A*) exhibits a direct correlation with *E*_*a*_, and its values also rise in tandem with the concentration of **QA** derivatives, a pattern consistent with prior research^43^.

**Table 3:** Activation parameters as a function of **QA** derivatives concentration at 298 K.

Levelling agent	C*10^3^ (mol/L)	Ea (kJ/mol)	A	ΔH* (kJ/mol)	− ΔS* (J/mol.K)	ΔG* (kJ/mol)
***m*-CH**_**3**_**-QA**	**0.00**	**15.22**	**8.90E + 02**	**12.74**	**196.76**	**71.38**
	0.33	23.85	1.91E + 04	21.37	171.25	72.40
	0.55	25.68	3.75E + 04	23.21	165.65	72.57
	0.77	30.45	2.23E + 05	27.97	150.82	72.91
	0.97	34.89	1.18E + 06	32.42	136.98	73.24
	1.18	39.38	6.19E + 06	36.90	123.21	73.61
	1.38	40.20	8.01E + 06	37.72	121.06	73.80
	1.58	41.00	1.03E + 07	38.52	119.01	73.98
	1.78	42.49	1.72E + 07	40.01	114.73	74.20
	2.07	42.82	1.86E + 07	40.34	114.04	74.33
	2.54	49.36	2.26E + 08	46.88	93.28	74.68
	3.00	52.02	5.92E + 08	49.54	85.29	74.96
	3.43	60.58	1.37E + 10	58.10	59.17	75.74
***o*-Cl-QA**	**0.00**	**15.22**	**8.90E + 02**	**12.75**	**196.76**	**71.38**
	0.33	24.92	3.13E + 04	22.44	167.15	72.25
	0.55	25.99	4.51E + 04	23.52	164.13	72.43
	0.77	28.58	1.15E + 05	26.10	156.37	72.70
	0.97	33.51	7.16E + 05	31.03	141.13	73.08
	1.18	35.19	1.28E + 06	32.71	136.28	73.32
	1.38	40.21	8.61E + 06	37.73	120.45	73.62
	1.58	39.25	5.42E + 06	36.78	124.31	73.82
	1.78	39.77	6.19E + 06	37.29	123.20	74.01
	2.07	39.25	4.80E + 06	36.77	125.32	74.11
	2.54	43.64	2.56E + 07	41.16	111.39	74.35
	3.00	44.31	3.06E + 07	41.84	109.92	74.59
	3.43	51.33	4.27E + 08	48.85	88.00	75.07
***p*-(CH**_**3**_**)**_**2**_**N-QA**	**0.00**	**15.22**	**8.90E + 02**	**12.75**	**196.76**	**71.38**
	0.33	25.64	4.34E + 04	23.17	164.43	72.17
	0.55	26.92	2.99E + 04	24.44	162.09	72.75
	0.77	25.05	2.96E + 04	22.57	167.62	72.52
	0.97	26.84	5.49E + 04	24.36	162.49	72.78
	1.18	29.72	1.61E + 05	27.24	153.53	72.99
	1.38	34.37	9.38E + 05	31.89	138.89	73.29
	1.58	31.69	3.07E + 05	29.21	148.16	73.37
	1.78	31.50	2.71E + 05	29.03	149.22	73.49
	2.07	31.89	3.00E + 05	29.41	148.36	73.62
	2.54	31.54	2.55E + 05	29.06	149.72	73.68
	3.00	29.62	1.14E + 05	27.14	156.43	73.76
	3.43	30.07	1.29E + 05	27.59	155.41	73.90

Table 3 clarifies the activation parameters (ΔH^*^, ΔS^*^, ΔG^*^) for our elaborated work, which are temperature-dependent for the metal dissolution process, corresponding to ${I}_{l}$ according to the perspective transition state theory, Eq. (3) is computed as follows:

3 $${I}_{l}= \frac{RT}{Nh}\text{exp}\left(\frac{{ \Delta S}^{*}}{R}\right)\text{exp}\left(-\frac{{\Delta H}^{*}}{RT}\right)$$

whereas the activation enthalpy is ΔH*, the activation entropy is symbolized by ΔS^*^, Planck’s constant is denoted by *h*, and *N* stands for Avogadro’s number. Identification of ΔH^*^ is represented by E_a_ and ΔH^*^ verifications as follows:

4 $$\Delta {\text{H}}^{*} = {\text{ E}}_{{\text{a}}} - {\text{RT}}$$

The enthalpy change is positive, as shown by the Table’s values, showing endothermic activity during the C-steel dissolution process with and without adding **QA** derivatives in 8M H_3_PO_4_^44^. Moreover, the calculated ΔS* displayed negative values, which are a good reference for the rate-determining step. According to the data, an active complex is produced via a process that involves association rather than separation. The substantially bigger negative entropy shift for each inhibitor proves this. As a result, disorder (randomness) decreases as the process progresses^45^.

In addition to the mentioned activation criteria, one more main activation parameter is estimated from the Arrhenius plot, which is the change in energy of activation ΔG^*^ from the ensuing relation:

5 $$\Delta {\text{G}}^{*} = \, \Delta {\text{H}}^{*} - {\text{T }}\Delta {\text{S}}^{*}$$

Table 3 shows that the limitation in increasing ΔG* magnitudes is detected. In other words, after adding inhibitors, the protected systems possess more positive ΔG^*^ than those in 8M H_3_PO_4_ only. This demonstrated that the activated complex is less stable under the influence of QA compounds than in its absence^46,47^.

### Isokinetic relationship (IKR)

The dissolution of C-steel in different **QA** concentrations showed an enthalpy-entropy compensation (EEC) and ***IKR*** that are related to the variation in the enthalpy of activation ΔH* or the entropy of activation ΔS*, or even both, which could be the source of rate inconstancy in a reaction series. The activation enthalpies (ΔH*) and entropies (∆S*) for the EP of QA through dissolution are linearly related, as clarified in Eq. (6):

6 $$\Delta {\text{H}}^{*} = \beta \Delta {\text{S}}^{*} + {\text{ constant}}$$

**Fig. 4:**
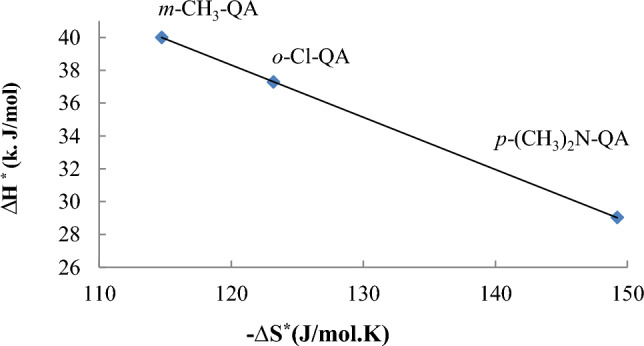
The isokinetic relationship for **QA**.

Experimentally, *β* can be determined as the slope of ΔH* vs. ΔS*, which is found to be linear (Fig. 4) and can respectively be described by ΔH* = − 0.3183 ΔS* + 76.513 with *R*^*2*^ = 1. Such behavior indicates that the EP reaction follows a common rate-determining step for both the free and the inhibited systems. The evaluated isokinetic temperature *β* was 318.3 K, i.e., higher than the experimental temperature, *T*_exp_ = 298 K. When *β* > *T*_exp_, the controlling factor is ΔS*, which confirms that the EP reaction is an assertion on the entropy-controlled reaction. Conjointly, the considerable linearity of the isokinetic relationship for **QA** asserts that their involvement plays an indispensable role in the rate of EP reactions. In other words, all **QA** undergo the same mechanism during EP.

### Modeling adsorption

Adsorption isotherm models (*Langmuir, Flory–Huggins, and kinetic*) are a pivotal tool for comprehending the inhibition mechanism in EP. They were used to fit the Galvanostatic data and analyze the **QA** derivative adsorption on the C-steel/medium interface in H₃PO₄. The data confirmed that **QA** forms a compact protective layer, suppressing EP by blocking active sites. Surface coverage (θ) was calculated at equilibrium, showing concentration-dependent adsorption at a uniform temperature^48^.

**Fig. 5:**
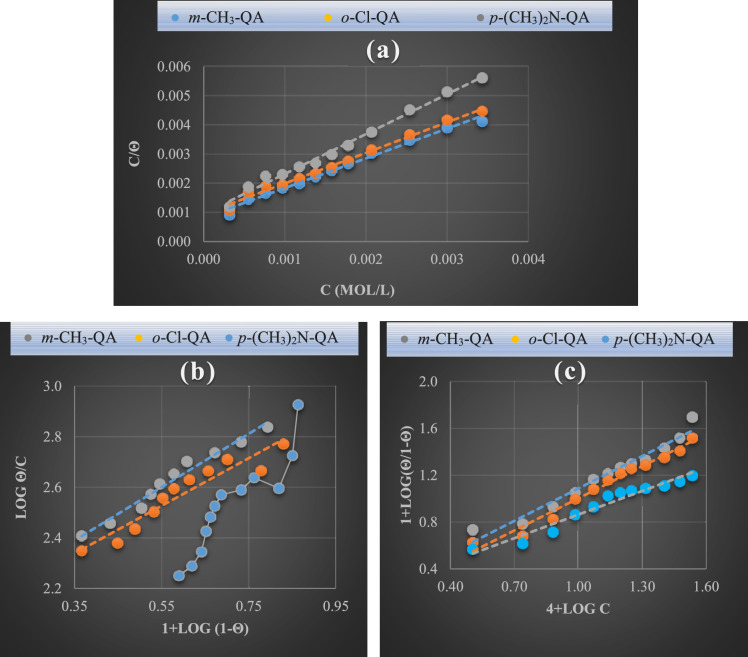
Adsorption models for **QA** derivatives on C-steel surface at 298K.

Figure 5 displays the fitting results. The following lists the pertinent expressions:

- ***Langmuir isotherm*:**
  7 $$\frac{C}{\uptheta }=C+\frac{1}{{K}_{ads}}$$
- ***Flory–Huggins isotherm*:**
  8 $$log \frac{\theta }{c}=\text{log xK}+\text{ x log}(1-\theta )$$
- ***Kinetic adsorption isotherm:***
  9 $${\varvec{l}}{\varvec{o}}{\varvec{g}}\frac{ \theta }{1-\theta }=logK{\prime}+y logc$$

where *θ* denotes the coverage of the metal surface, which is obtained from the electrochemical fitting data, *C* is the **QA** inhibitor contain (mol/L); *K*_*ads*_ is the adsorption equilibrium constant, *x* is the number of organic molecules replacing water molecules during the adsorption process, *K* is binding constant indicates the binding force between **QA** derivatives and steel surface, 1/y represents the number of active sites which can be occupied by one inhibitor molecule, and *K* is the binding constant relating to constant Kˋ utilizing the relation K = Kˋ^**1/y**^.

Figure 5a and Table 4 indicate that while all compounds showed good linear correlation (R^2^), only ***m*-CH3-QA** and ***o*-Cl-QA** had slopes of 1, fitting the Langmuir model by forming a monolayer on the C-steel surface. In contrast, ***p*-(CH3)2N-QA** deviated with a slope > 1, suggesting non-compliance with Langmuir adsorption behavior^49,50^.

**Table 4:** Fitting parameters of adsorption isotherm models for **QA** derivatives at 298 K.

Levelling agent	Parameters										
	Langmuir			Flory Huggin’s			Kinetic adsorption isotherm				
	slope	K	ΔG^o^_ads_	x	K	ΔG^o^_ads_	Kˋ	K_ads_	1/y	R^2^	ΔG^o^_ads_
***m*-CH**_**3**_**-QA**	1.02	1250.00	− 27,618.19	1.06	1128.39	− 27,364.61	706.64	1239.53	1.08	0.9622	− 27.59
***o*-Cl-QA**	1.05	1111.11	− 27,326.38	0.94	948.19	− 26,933.53	554.88	1004.79	1.09	0.9749	− 27.07
***p*-(CH**_**3**_**)**_**2**_**N-QA**	1.37	1111.11	− 27,326.38	–	–	–	76.15	626.35	1.48	0.9512	− 25.91

Investigating Fig. 5b, a good straight line is produced for ***m*-CH**_**3**_**-QA** and ***o*-Cl-QA** with high correlation coefficient values (R^2^), as recorded in Table 4. As well, values of *x* are close to unity, suggesting that one molecule of ***m*-CH**_**3**_**-QA** and ***o*-Cl-QA** displaces a water molecule at a definite temperature. Conversely, ***p*-(CH**_**3**_**)**_**2**_**N-QA** compounds don’t fit the model as in Fig. 5b^51,52^.

Equation (9) has been schemed as shown in Fig. 5c, which yields a straight line with a high R^2^ value. The slope *y* and the values of *K* and *K*’ are assembled in Table 4, affirmed that:

I. Values of *1/y* for ***m*-CH**_**3**_**-QA** and ***o*-Cl-QA** show uniformity so that an inhibitor molecule can occupy one active site. Meanwhile, the compound ***p*-(CH**_**3**_**)**_**2**_**N-QA** can reside at more than one active site on the metal surface as the value of *1/y* exceeds unity.
II. The considerable values of the binding constant *K*_*ads*_ reveal the interaction intensity between inhibitor molecules and the carbon steel surface.

To achieve a comprehensive understanding of the adsorption mechanism of **QA** derivatives on C-steel, the mathematical relationship between *K*_*ads*_ and the adsorption-free energy *ΔG*^*o*^_*ads*_ can be utilized to calculate *ΔG*^*o*^_*ads*_, as outlined in the corresponding Eq. (10) for **QA** derivatives:

10 $${K}_{ads.}=\frac{1}{55.5}\text{exp}\left(-\frac{{\Delta G}_{ads.}^{o}}{RT}\right)$$

Knowing that *T* is the absolute temperature, and 55.5 is the water concentration (mol/L) in solution^53^. By inspecting Table 4, the values of ${\Delta G}_{ads}^{^\circ }$ range from − 27.07 to − 25.91 kJ/mol, elucidating that the **QA** compounds are adherent to hybrid physisorption-chemisorption mechanism. In addition, the large negative values are indication for the spontaneity of the adsorption reaction. This ensures the congestion of the active sites on carbon steel surface has been built up by **QA** coating preventing EP^43^. Remarkably, the predominant value of ${\Delta G}_{ads}^{^\circ }$ for ***m*-CH**_**3**_**-QA** notarizes formation of protective layer on C-steel and hence, more excessive inhibition efficiency than ***o*-Cl-QA** and ***p*-(CH**_**3**_**)**_**2**_**N-QA,** respectively, as exhibited in Table 4^54^.

#### Dubinin-Radushkevich isotherm model

An adscititious model that is well known to diagnose the adsorption process is the Dubinin-Radushkevich model^55^, which the following Eq. can identify:

11 $$\text{ln}\theta =\text{ln}{\theta }_{max}-a{\delta }^{2}$$

Where the maximum surface coverage is symbolized by ${\varvec{\theta}}$ and the Polanyi potential ${\varvec{\delta}}$ given by:

12 $$\delta =RT\text{ln}\left(1+\frac{1}{{C}_{inh}}\right)$$

**Fig. 6:**
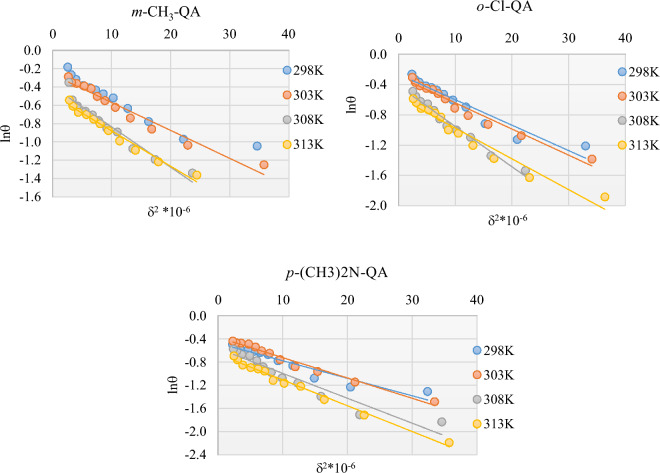
Dubinin-Radushkevich plots for EP of C-steel in the presence of **QA** derivatives.

In the previous Equation, ${C}_{inh}$ is the concentration of **QA** compound in g/L. Parameter ***a*** can be identified from plots of $\mathit{ln}\theta$ against $\delta$^2^ as cleared in Fig. 6.

Consequently, the mean adsorption energy *E*_*m*_, which is defined as the transfer energy of *1* mol of adsorbate from infinity (bulk solution) to the surface of the adsorbent for the related temperature, can be introduced as:

13 $${E}_{m}=\frac{1}{\sqrt{2a}}$$

The manner of adsorption can be inferred from the magnitude of *E*_*m*_^56^: inferior to 8 kJmol^-1^ is an indication for physiosorption, while that superior to 8 kJ mol^−1^ is a suggestion for chemisorption. Observing Table 5, values of *E*_*m*_ are affirmations for the physical type.

**Table 5:** Parameters of the Dubinin-Radushkevich isotherm.

Levelling Agent	T(K)	R^2^	a (kJ^−2^mol^2^)	θ_max_	E_m_(kJmol^−1^)
***m*-CH**_**3**_**-QA**	298	0.9188	0.0278	0.7976	4.24
	303	0.9617	0.0305	0.6296	4.05
	308	0.9422	0.0441	0.5829	3.37
	313	0.9743	0.0386	0.6103	3.59
***o*-Cl-QA**	298	0.9263	0.0335	0.7659	3.86
	303	0.9678	0.0345	0.7426	3.81
	308	0.9787	0.0534	0.6578	3.06
	313	0.9503	0.0406	0.5662	3.51
***p*-(CH**_**3**_**)**_**2**_**N-QA**	298	0.9153	0.0302	0.6219	4.07
	303	0.9799	0.0351	0.6885	3.77
	308	0.9223	0.0430	0.5695	3.41
	313	0.9878	0.0446	0.5164	3.35

Free energy of adsorption (*Q*_*ads*_), which is attained from the kinetic thermodynamics model, is utilized to focus more on the adsorption process, and can be introduced by Eq. (14)

14 $${Q}_{ads}=2.303\text{R }\left[\left(\frac{{\theta }_{2}}{1-{\theta }_{2}}\right)-\text{log}\left(\frac{{\theta }_{1}}{1-{\theta }_{1 }}\right)\right]\frac{{T}_{2 }{T}_{1}}{{T}_{2}-{T}_{1}}$$

whereas, ${\theta }_{1}$ and ${\theta }_{2}$ are the grades of surface coverage at temperatures ${T}_{1}$ and ${T}_{2}$ 298 and 308 sequentially. By analyzing Table 6, the negative magnitudes of *Q*_*ads*_ further confirm the exothermic nature of the adsorption process of **QA** on the C-steel surface, with higher temperatures leading to weaker adsorption and lower inhibition performance. However, **QA** derivatives performs efficiently as protective organic compounds throughout a hybrid physisorption-chemisorption mechanism^57^.

**Table 6:** Heat of adsorption from Kinetic-thermodynamic adsorption isotherm on C-steel surface in 8M H_3_PO_4_ at various concentrations of **QA**.

C(mol/L)*10^–3^	*m*-CH_3_-QA	*o*-Cl-QA	*p*-(CH_3_)_2_N-QA
	Q_ads_(kJ/mol)	Q_ads_(kJ/mol)	Q_ads_(kJ/mol)
**0.332**	− 50.970	− 40.951	− 38.561
**0.549**	− 47.307	− 42.737	− 41.354
**0.763**	− 50.889	− 48.250	− 33.838
**0.973**	− 59.200	− 52.078	− 36.144
**1.18**	− 57.236	− 55.642	− 37.619
**1.38**	− 62.598	− 60.246	− 42.942
**1.58**	− 60.762	− 57.598	− 35.762
**1.78**	− 54.217	− 52.080	− 24.668
**2.07**	− 53.400	− 44.641	− 15.088
**2.54**	− 62.247	− 50.780	− 15.855
**3.00**	− 65.828	− 48.967	− 15.261
**3.43**	− 56.980	− 55.455	− 15.628

### Surface study

#### Atomic force microscope (AFM)

**Fig. 7:**
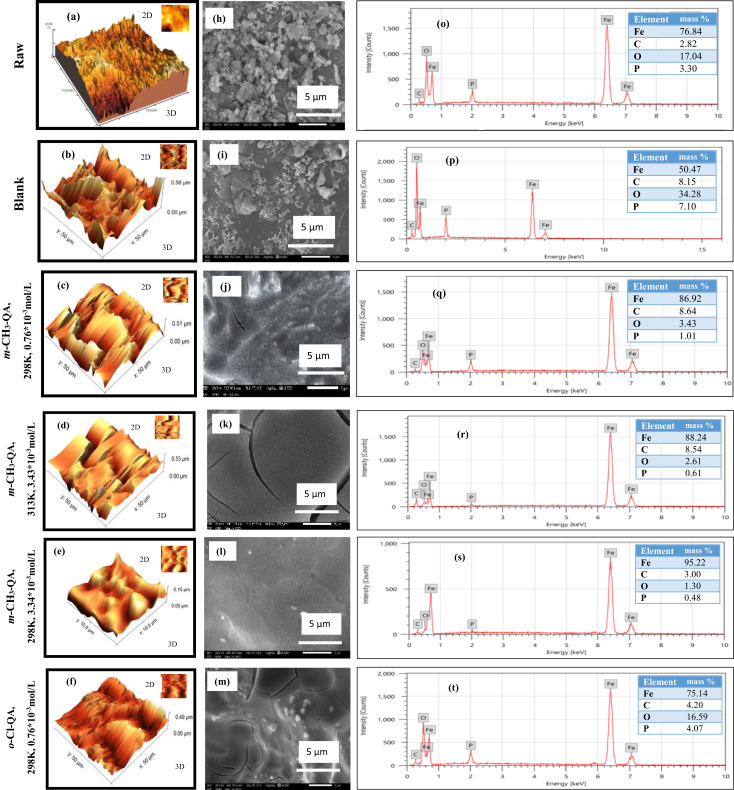

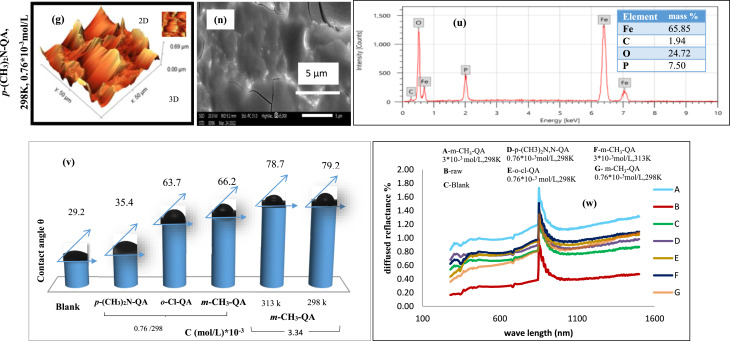
3D, 2D AFM images (**a**–**g**), SEM images (**h**–**n**), EDX analysis (**o**–**u**), contact angle (**v**), and reflectance for C-steel specimens without and with **QA** derivatives (**w**).

Acquiring quantitative details about surface topography at *µm* scales to assess qualitative outcomes from SEM requires the use of *AFM* (Sharma and Kumar, 2021), which is considered a prolific technique to recognize the consequences of absence and attendance for **QA** derivatives during EP^8,43,49,58,59^. As illustrated in Fig. 7a–g, three-dimensional (3D) and two-dimensional (2D) *AFM* images show the surface structure of 3 kinds of C-steel, which are raw samples, electropolished samples in 8M H_3_PO_4_, and protected specimens after treatment with **QA** derivatives under different conditions. In accordance with Table 7, average roughness (*Ra*), peak-to-valley ratio (*PV*), and mean square roughness (*Rq*) were stated and trended similarly.

**Table 7:** Surface roughness parameters of C-steel surfaces before and after EP in 8 M H_3_PO4 solutions without and with **QA** derivatives.

Parameters (µm)	Raw	Blank	*p*-(CH_3_)_2_N-QA 298K 0.76*10^–3^ mol/L	*o*-Cl-QA 298K 0.76*10^–3^ mol/L	*m*-CH_3_-QA		
					298K, 0.76*10^–3^ mol/L	313K, 3.43*10^–3^ mol/L	298K 3.43*10^-3^mol/L
**Ra ± SD**	540.20 ± 12.3	418.19 ± 10.5	14.15 ± 0.6	8.16 ± 0.3	7.95 ± 0.3	7.32 ± 0.3	4.67 ± 0.21
**Rq ± SD**	441.10 ± 9.8	556.63 ± 11.2	21.35 ± 0.9	11.09 ± 0.5	10.76 ± 0.4	9.22 ± 0.4	6.98 ± 0.35
**PV ± SD**	61.20 ± 2.1	54.63 ± 1.8	49.14 ± 2.0	42.05 ± 1.7	39.79 ± 1.6	36.62 ± 1.5	28.07 ± 1.2

Looking over Fig. 7a and Table 7, the blank specimen of the C-steel surface seems distorted with the maximal values of *Ra* (540.2 µm) and *PV* ratio (61.20µm). These magnitudes were subtracted to (418.19 µm) and (54.632 µm) respectively, subsequent to EP in an aggressive medium. Surface distortion is reduced relatively compared to blank as in Fig. 7b. Hence, it is noticeable that all are at minimum values for C-steel after treatment with ***p*-(CH**_**3**_**)**_**2**_**N-QA**, ***o*-Cl-QA**, and ***m*-CH**_**3**_**-QA** under various conditions**.** Remarkably, surface protrusions and unevenness diminish after adding ***m*-CH**_**3**_**-QA** in contrast to ***p*-(CH**_**3**_**)**_**2**_**N-QA** and ***o*-Cl-QA**, as in Fig. 7c,f,g. This authenticated the order of IE% as the following: ***m*-CH**_**3**_**-QA** > ***o*-Cl-QA** > ***p*-(CH**_**3**_**)**_**2**_**N-QA**. Also, the surface will be improved by alternating temperature and concentration in the presence of ***m*-CH**_**3**_**-QA** (Fig. 7c–e), which could refurbish surface topography, resulting in diminishing the *Ra* and *PV* ratio as shown in Table 7. These results confirm that **QA** derivatives can hinder the anodic dissolution reaction in acidic media.

#### Morphological inspection utilizing (SEM)

SEM is a beneficial and stipulated method for C-steel surface inspection to assure the rate of EP results^43,44,49^. Hence, Fig. 7h–n exhibits images of SEM for C-steel specimens under various conditions. This Figure demonstrates that the surface of the raw sample is extremely damaged, with serious roughness accompanied by pits and cavities (Fig. 7h). This surface roughness decreases gradually after EP in 8M H_3_PO_4_ at 298K, and the C-steel surface becomes more homogeneous, as shown in Fig. 7i for the blank sample.

Figure 7j–l shows the typical SEM cross-section image of the C-steel sample under different concentrations of ***m*-CH**_**3**_**-QA**. The results reveal that discriminating between images for lessening the concentration of ***m*-CH**_**3**_**-QA** from 3.43*10^-3^mol/L to 0.76*10^-3^mol/L allowed deformation of the inspected specimen where the surface became less homogeneous and more irregular with a subsequent heterogeneous distribution. Consequently, affirmation of concentration change is prominent in altering surface morphology.

SEM images of C-steel samples after EP in 3.34 × 10^-3^mol/L ***m*-CH**_**3**_**-QA** at different temperatures are displayed in Fig. 7k,l. The surface is extensively impacted by temperature, becoming remarkably smooth and perfectly even with a mirror-like structure, as shown in Fig. 7l. The enhancement of the C-steel surface structure indicates the formation of a protective layer on the surface in a regular way. This could proceed from the adsorption of the *–CH*_*3*_ functional group on a metal surface via electron donation or attributed to the enmeshment of **QA** derivative molecules with the active sites on C-steel, resulting in diminished interaction between the acidic medium and C-steel, and consecutive an outstanding inhibition effect has been manifested. Nevertheless, elevating the temperature in our study influenced the even morphology as progressive damage was released with teeny peaks and valleys and some cracks. It could arise from the desorption of ***m*-CH**_**3**_ molecules during EP at elevated temperatures.

Conversely, treating the C-steel surface with 0.76*10^-3^mol/L ***o*-Cl-QA** at 298K modified its morphology (Fig. 7m). In contradistinction to the image belonging to ***m*-CH**_**3**_**-QA** under the same conditions, the surface roughness appeared in the form of micrometer-sized holes with a random distribution associated with some irregularities. This could be a consequence of the electron withdrawing feature of the *–Cl* function group that subsides the protective layer formation during EP. Furthermore, the preceding EP of C-steel in 8M H_3_PO_4_ and 0.76*10^-3^mol/L **QA** derivative with a bulk functional group as ***p-N, N****-*dimethyl has a prominent command in the un-uniform surface morphology (Fig. 7n). Exploring the image of ***p*-(CH**_**3**_**)**_**2**_**N-QA**, a grain-like structure arose with unequal size among obvious irregularities and surrounded by them, developing out of the steric hindrance of *–N, N*-dimethyl functional group. This could diminish its blocking of C-steel active sites and inhibition retardation.

These findings utterly validate the AFM image results and their widespread praise. The sections on reflectance and water contact angle measurements that follow will provide more evidence that scanning electron microscope pictures support roughness measures.

#### EDX analysis

With the assistance of EDX outcomes, it is possible to identify the chemical composition and structure of the C-steel surface we studied. The spectrum of EDX for the C-steel specimen during EP in 8M H_3_PO_4_ before and after treatment with **QA** has been illustrated in Fig. 7o–u. In our study, the revealed signals for *Fe* were sizable, while further elements, such as *O*, *C*, and *P*, were also detected. Examining the Tables illustrated in Fig. 7, the mass% of the EP C-steel in 8M H3PO4 under various conditions is contrasted. It is ascertained that the mass percentages of *Fe* are in escalation after treatment with **QA** (Fig. 7q–u)rather than that of *Fe* in aggressive medium only (Fig. 7p). Quite the contrary, the mass% of *O* and *P* in de-escalation proposed the limitation in active sites of EP. This is due to the lower formation of iron oxide and the greater protection provided by **QA**. However, the mass% of *C* atoms is in growth, which can be assumed from the adsorption of some **QA** molecules. This was a testimony to the molecular barrier consistency at the interface between the electrode and electrolyte via QA molecules during EP, which restrained the Fe_3_(PO)_4_ complex developing^57,59,60^.

#### Water contact angle (WCA)approach

One specific method to assess whether an additive exhibits hydrophobic or hydrophilic behavior while protecting the C-steel surface from anodic dissolution is the water contact angle (WCA) measurement^61^. As reported in the literature^58^, iron oxides are typically formed during EP under aggressive conditions and exhibit hydrophilic characteristics. As shown in Fig. 7v, the C-steel specimen in the blank solution displayed the lowest *WCA* value (29.9°), indicating that hydrophilic EP products and rust covered the sample surface.

In contrast, the presence of **QA** derivatives in the aggressive medium altered the surface’s hydrophilicity. The *WCA* values increased progressively to 35.4°, 63.7°, 66.2°, and up to 79.2°, corresponding to 0.76 × 10⁻^3^ mol/L of ***p*-(CH**_**3**_**)**_**2**_**N-QA**, 0.76 × 10⁻^3^ mol/L of ***o*-Cl-QA**, and 0.76 × 10⁻^3^ mol/L to 3.43 × 10⁻^3^ mol/L of ***m*-CH₃-QA**, respectively (Fig. 7v).

Based on FT-IR analysis, the presence of *C–C* and *C*=*C* bonds in all aromatic **QA** derivatives contributes to their surface adsorption. The aggregation of these adsorbed molecules forms a coherent hydrophobic protective layer on the C-steel surface, effectively blocking the penetration of the EP solution to the metal–electrolyte interface^61^.

Additionally, changes in EP conditions influence the WCA values. As shown in Fig. 7v, increasing the temperature from 298 to 313 K and raising the concentration of ***m*-CH₃-QA** from 0.76 × 10⁻^3^ to 3.43 × 10⁻^3^ mol/L enhanced the hydrophobicity of the adsorbed protective layer. This is evident from the plots: higher temperature resulted in a lower *WCA* (78.7°), indicating reduced inhibition efficiency. Conversely, increasing the concentration led to a higher *WCA* (79.2°), indicating stronger adsorption and the formation of a more effective protective layer, a finding consistent with the SEM observations.

#### Reflectance

The aggressive EP has a drastic influence on brightening and smoothing C-steel surfaces. The brightness and surface roughness are inversely correlated to a considerable extent^32,50^. As illustrated in Table 7, the gleam of C-steel surfaces with **QA** grows as the roughness diminishes. This clarifies that the more scattered the light, the less gleaming the surface.

In consonance with this inquiry, submersion of the specimen in 8 M H_3_PO_4_ solution elevates reflectance progressively. At the same time, the minimal value is noticed for the raw specimen, as shown in Fig. 7w. The apparition of fragile films on the C-steel surface could result in a distinct variation in luster value. **QA**-treated specimens showed improved reflectance% relative to the blank. As a consequence, the inherent characteristics of the C-steel surface have been modified.

Two factors could elevate the reflectance%, which are inhibitor concentration and the rate of its adsorption. As the adsorption rate increases, the luster of the surface increases, becoming more pronounced with inflated **QA** concentrations. Figure 7w depicts reflectance plots for EP surfaces in a blank electrolyte and multiple inhibitor concentrations. Various samples showed a reduction as the inhibitor type changed^50^.

#### AAS measurements

In this study, the iron ions (*Fe*^2+^) generated by C-steel corrosion in the H_3_PO_4_ environment were quantified by atomic absorption spectroscopy. The absorbance percentage inhibition efficiency (%€AAS) of the **QA** was obtained using Eq. (15). Based on the tabulated data (Table 8), the findings of the AAS showed that *Fe*^2+^ concentration was lowered in the corrosive solution by increasing the inhibitor concentration, decreasing the temperature, and using different **QA** derivatives. As a function of corrosion rate, *Fe*^2+^ was modeled in this study, suggesting that a rise in iron ion concentration causes an increase in corrosion rate and vice versa. This reveals that the inhibition efficiency of the various techniques follows the order***m-*CH**_**3**_ $>$ ***o-*Cl** > ***p-N, N***^62^.

**Table 8:** Demonstrates the impact of QA concentrations, types, and temperatures on *Fe*^*2*+^ concentrations using the AAS method.

Samples	[Fe^2+^] (mg.L^−1^)	Signal Absorbance	%€AAS
Iron + 8 M H_3_PO_4_ (298 K)	101.30	0.61700	–
Iron + 8 M H_3_PO_4_ (303 K)	111.10	0.68019	–
Iron + 8 M H_3_PO_4_ (308 K)	123.50	0.96100	–
Iron + 8 M H_3_PO_4_ (313 K)	132.90	1.39530	–
Iron + 8 M H_3_PO_4_ + 3.00 × 10^–3^ *m*-CH_3_-QA (298 K)	19.37	0.17590	80.88
Iron + 8 M H_3_PO_4_ + 3.00 × 10^–3^ *o*-Cl-QA (298 K)	29.18	0.23580	71.19
Iron + 8 M H_3_PO_4_ + 3.00 × 10^–3^ *p*-(CH_3_)_2_N-QA (298 K)	43.78	0.29306	56.78
Iron + 8 M H_3_PO_4_ + 0.76 × 10^–3^ *m*-CH_3_-QA (298 K)	63.19	0.36078	37.62
Iron + 8 M H_3_PO_4_ + 3.00 × 10^–3^ *m*-CH_3_-QA (298 K)	19.37	0.17590	80.88
Iron + 8 M H_3_PO_4_ + 3.00 × 10^–3^ *m*-CH_3_-QA (298K)	19.37	0.17590	80.88
Iron + 8 M H_3_PO_4_ + 3.00 × 10^–3^ *m*-CH_3_-QA (303 K)	35.62	0.44300	67.94
Iron + 8 M H_3_PO_4_ + 3.00 × 10^–3^ *m*-CH3-QA (308 K)	49.11	0.51309	60.23
Iron + 8 M H_3_PO_4_ + 3.00 × 10^–3^ *m*-CH_3_-QA (313 K)	67.03	0.79100	49.56

15 $$\%  {\text{AAS = }}\left( {{\text{1}} - \frac{{{\text{C}}_{{{\text{QA}}}} }}{{{\text{C}}_{{{\text{blank}}}} }}} \right) \times {\text{100}}.$$

#### DFT studies of QA

**Fig. 8:**
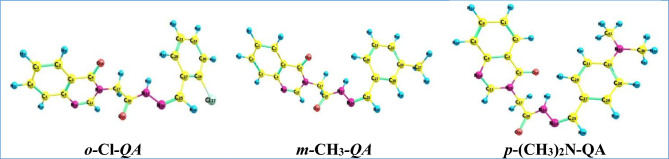
The optimized geometries of the studied compounds **QA**.

Density Functional Theory (DFT) calculations were carried out to obtain the optimized geometrical structures and key thermodynamic descriptors of the **QA** derivatives (***o-Cl-QA*, *m-CH***_***3***_***-QA***, and ***p-(CH***_***3***_***)***_***2***_***N-QA***), using the B3LYP/6-311G(d,p) method. The absence of imaginary frequencies confirmed the stability of all optimized structures, and the results are summarized in Fig. 8 and Table 9.

**Table 9:** Estimated thermal parameters, polarizability, and dipole moment for the investigated series, **QA**_**.**_

Compound	ZPE (kcal/mol)	Thermal energy (kcal/mol)	Enthalpy (kcal/mol)	Gibbs free energy (kcal/mol)	Entropy (Cal mol.K)	Polarizability (α) Bohr^3^	Dipole moment (D)
*o-*Cl*-QA*	175.838	188.046	188.638	143.110	152.707	287.492	8.646
*m-*CH_3_*-QA*	199.537	212.088	212.680	166.261	155.695	293.311	8.276
*p*-(CH_3_)_2_N-QA	228.506	242.575	243.167	194.212	164.197	348.680	11.688

The zero-point energy (ZPE) and other thermodynamic parameters, such as thermal energy, enthalpy, and Gibbs free energy, are known to influence the adsorption behavior of inhibitor molecules on metal surfaces. The compound ***o-*Cl*-*QA**, containing an electron-withdrawing chloro group, exhibited the lowest ZPE and thermal values, indicating reduced thermal reactivity and limited adsorption capability, consistent with its lower experimental inhibition efficiency.

Conversely, ***p-(CH3)₂N-QA*** showed the highest ZPE, dipole moment, and polarizability due to the bulky dimethylamino substituent, which may enhance polar interactions. However, its inhibition performance was not the highest, likely because the bulky group causes steric hindrance, reducing its ability to adsorb effectively on the metal surface.

***m-CH3-QA*** demonstrated the most favorable balance between electronic and thermal properties. Its moderate ZPE, relatively low dipole moment, and suitable polarizability, along with the electron-donating methyl group, enhance its ability to interact with the steel surface via electron donation and stable adsorption. This correlates well with its superior inhibition efficiency observed experimentally.

Overall, the DFT-based thermodynamic and electronic descriptors provide a consistent theoretical basis for explaining the experimental performance of the QA derivatives, highlighting ***m-CH3-QA*** as the most effective inhibitor in the series^63,64^.

#### Frontier molecular orbitals

The analysis of frontier molecular orbitals (FMOs), specifically the highest occupied molecular orbital (HOMO) and the lowest unoccupied molecular orbital (LUMO), offers valuable insights into the electronic properties and reactivity of the **QA** derivatives. A higher HOMO energy indicates a stronger ability to donate electrons. In comparison, a lower LUMO energy reflects an increased tendency to accept electrons, both of which are favorable for adsorption onto metal surfaces and effective corrosion inhibition.

The calculated orbital energies (Table 10) show that ***m-*CH**_**3**_***-*QA** possesses the highest HOMO energy (-6.34 eV), suggesting enhanced electron-donating ability toward the metal surface. In contrast, ***p*-(CH**_**3**_**)**_**2**_**N-QA** has the lowest HOMO energy and the largest HOMO–LUMO gap, which indicates reduced chemical softness and weaker adsorption interactions. While **o-Cl-QA** exhibits the lowest LUMO energy (− 1.76 eV), its overall performance is limited by a relatively larger energy gap compared to ***m*-CH**_**3**_**-QA**.

**Table 10:** Various quantum chemical parameters for the optimized structures of the studied molecules at B3LYP/6-311G(d,p) level of theory.

Parameter	*o-*Cl*-*QA	*m-*CH_3_*-*QA	*p*-(CH_3_)_2_N-QA
E (Hardness)	− 1484.9909	− 1064.7269	− 1159.3543
E_HOMO_ (eV)	− 6.5676	− 6.3380	− 6.8663
E_LUMO_ (eV)	− 1.7643	− 1.6351	− 1.6800
∆E (eV)	4.8032	4.7029	5.1864
IP (eV)	6.5676	6.3380	6.8663
EA (eV)	1.7643	1.6351	1.6800
χ (eV)	4.1660	3.9865	4.2732
µ (eV)	− 4.1660	− 3.9865	− 4.2732
η (eV)	2.4016	2.3515	2.5932
σ (eV^-1^)	0.4164	0.4253	0.3856
ω (eV)	3.6132	3.3793	3.5208

The energy gap (ΔE) reflects molecular reactivity, with smaller gaps corresponding to higher softness and better interaction with the metal surface. The observed trend ***m-*CH**_**3**_***-*QA** (4.70 eV) < ***o-*Cl*-*QA** (4.80 eV) < ***p*-(CH**_**3**_**)**_**2**_**N-QA** (5.18 eV) correlates well with the experimentally observed inhibition efficiencies^65,66^.

**Fig. 9:**
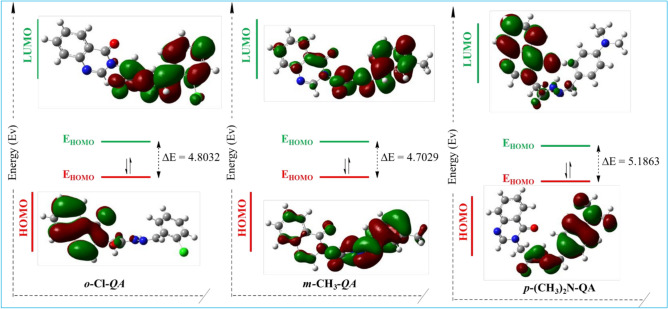
The estimated plots of Frontier molecular orbitals (FMOs) for compounds **QA**.

The spatial distribution of HOMO and LUMO, visualized in Fig. 9, further supports these findings. In ***m-*CH**_**3**_***-*QA**, both orbitals are localized on the benzylidene moiety, which is expected to be the primary adsorption center. In ***o-*Cl*-*QA**, the HOMO is spread over the quinazoline ring, while the LUMO is focused on the benzylidene unit. For ***p*-(CH**_**3**_**)**_**2**_**N-QA**, the reversed orbital distribution may contribute to its lower surface reactivity. The FMO analysis supports the superior inhibition performance of ***m-*CH**_**3**_***-*QA**, in line with experimental results, and confirms that favorable orbital energies and an appropriate electronic distribution are essential for effective molecular adsorption and corrosion inhibition^67,68^.

#### Chemical reactivity descriptors

Chemical reactivity descriptors derived from Density Functional Theory (DFT) provide crucial information about the stability, reactivity, and adsorption potential of the QA derivatives. Parameters such as electronegativity (χ), ionization potential (IP), hardness (η), softness (σ), and electrophilicity index (ω) are directly related to the HOMO–LUMO energies and can be used to predict inhibition performance. Among the three compounds (Table 10), ***m-*CH**_**3**_***-*QA** displayed the most favorable values, with the lowest hardness (η = 2.35 eV) and highest softness (σ = 0.43 eV^-1^), indicating enhanced polarizability and electron-donating ability. These characteristics favor stronger interactions with the metal surface, consistent with its superior experimental inhibition efficiency^4,69^.

Additionally, ***m-*CH**_**3**_***-*QA** showed the lowest electronegativity (χ = 3.99 eV) and lowest ionization potential (IP = 6.34 eV), both of which further support its enhanced reactivity and readiness to donate electrons to the metal surface. In contrast, ***p*-(CH**_**3**_**)**_**2**_**N-QA** exhibited the lowest softness and highest IP, indicating a reduced ability to interact electronically with the substrate, which aligns with its relatively poor inhibition performance. The order of the key descriptors aligns well with the experimental inhibition efficiencies: ***m-*CH**_**3**_***-*QA** > ***o-*Cl*-*QA** > ***p*-(CH**_**3**_**)**_**2**_**N-QA**, reaffirming the predictive reliability of these theoretical descriptors in evaluating corrosion inhibitor effectiveness^70^.

#### Molecular electrostatic potential (MEP)

The molecular electrostatic potential (MEP) maps offer valuable information about the charge distribution and reactive sites of the QA derivatives, which is critical for understanding their adsorption behavior on metal surfaces. Red regions in the MEP plots represent areas of high electron density (favorable for electrophilic attacks), while blue areas indicate low electron density (favorable for nucleophilic interactions).

**Fig. 10:**
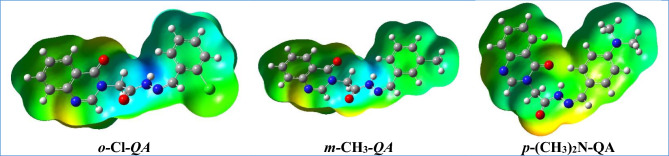
The molecular electrostatic potentials of the prepared **QA**.

As illustrated in Fig. 10, both ***m-*CH**_**3**_***-*QA** and ***o-*Cl*-*QA** exhibit red zones localized around electronegative atoms within the quinazoline ring, indicating potential adsorption sites. Blue regions are mainly found on the aliphatic parts of the azomethine group, suggesting lower reactivity in those areas. For ***p*-(CH**_**3**_**)**_**2**_**N-QA**, the red areas are more pronounced around nitrogen and oxygen atoms, while blue regions dominate the aromatic rings. These electrostatic patterns support the hypothesis that variations in substituent groups significantly influence the interaction of QA molecules with metal surfaces. The more localized and accessible the high electron density regions are, the more favorable the adsorption and, consequently, the inhibition performance, in agreement with experimental observations^71^.

#### Fukui indices and local dual descriptors

**Fig. 11:**
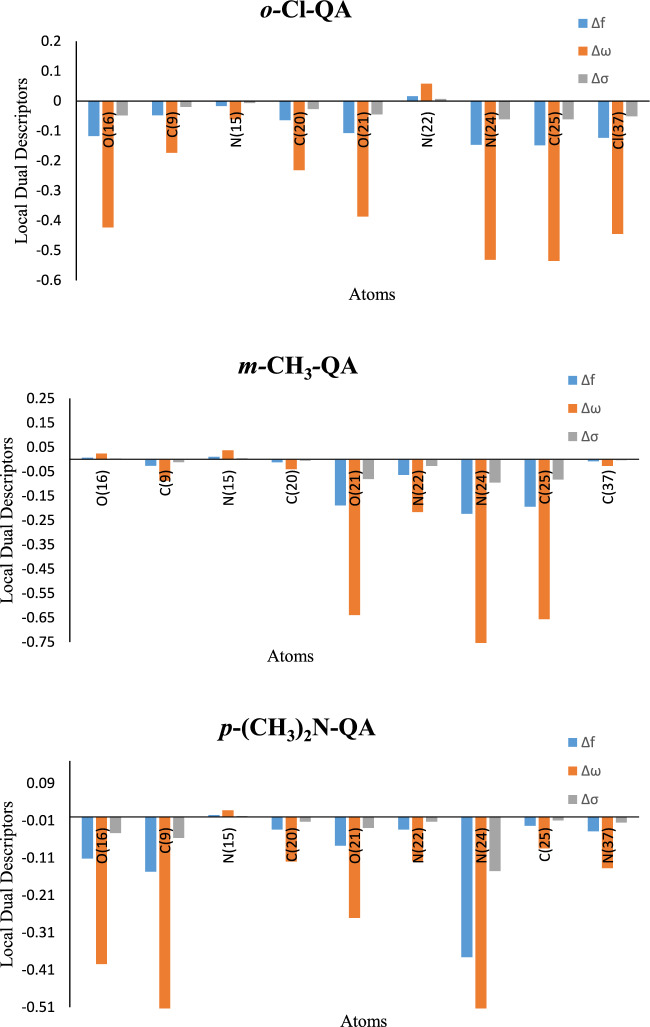
Graphical representation of Fukui function of **QA** derivatives.

Fukui indices and local dual descriptors provide detailed insights into the reactive behavior of **QA** derivatives by identifying potential electrophilic and nucleophilic attack sites at the atomic level. Using Mulliken charges, the condensed Fukui functions (f^+^, f⁻), local softness (σ^+^, σ⁻), and local electrophilicity (ω^+^, ω⁻) were calculated and are reported in the **supplementary data**. The differences (Δf, Δσ, and Δω) were also determined to pinpoint the preferred attack types on each atom. Atoms with positive Δ values are likely to undergo nucleophilic attacks, indicating regions of high electron deficiency and favorable interactions with electron-rich metal surfaces. Notably, atoms such as N(24), O(21), and C(25) in o-Cl-QA; N(22), N(24), and O(21) in ***m-*CH**_**3**_***-*QA**; and N(24) in ***p*-(CH**_**3**_**)**_**2**_**N-QA** show such reactivity, as visualized in Fig. 11. In contrast, atoms with negative Δ values are susceptible to electrophilic attacks, signifying areas of higher electron density. These include O(16) and C(9) in both ***o-*Cl*-*QA** and ***m-*CH**_**3**_***-*QA**, and O(16), C(9), and N(15) in ***p*-(CH**_**3**_**)**_**2**_**N-QA**. These localized electronic properties offer a deeper understanding of how different substituents modulate the electron distribution in the **QA** molecules, thus influencing their adsorption orientation and reactivity toward the metal surface. The Fukui analysis supports the experimental inhibition efficiency trend, confirming that the most reactive sites align with the observed inhibitory behavior^72^.

$${f}_{k}^{+}= {\rho }_{k}\left(N+1\right){\rho }_{K}\left(N\right) (nucleophilic attack)$$

$${f}_{k}^{-}= {\rho }_{k}\left(N\right){\rho }_{K}\left(N-1\right) (electrophilic attack)$$

16 $$\Delta f=\left({f}_{k}^{+}\right)--{(f}_{k}^{-})$$

$$\Delta\upomega =\left({\upomega }_{k}^{+}\right)--{(\upomega }_{k}^{-})$$

$$\Delta \sigma =\left({\sigma }_{k}^{+}\right)--{(\sigma }_{k}^{-})$$

where the electronic density site *ρ* is the number of electrons (*N*) at site k in a molecule.

Finally, the DFT-derived chemical reactivity descriptors, MEP analysis, and Fukui indices collectively support the experimental findings. For instance, the superior inhibition efficiency observed experimentally for ***m-*CH**_**3**_***-*QA** is in excellent agreement with its lower hardness (η), higher softness (σ), lower electronegativity (χ), and lower ionization potential (IP), all of which indicate a stronger tendency for electron donation and adsorption onto the metal surface. Furthermore, the Fukui analysis identifies nucleophilic and electrophilic reactive sites that correspond well with the proposed adsorption centers inferred from the electrochemical measurements. This theoretical–experimental alignment strengthens the proposed mechanism of adsorption and inhibition, confirming that ***m-*CH**_**3**_***-*QA** is the most effective corrosion inhibitor among the studied compounds.

## Conclusion

- A novel series of Quinazolin Schiff-base derivatives (**QA**) was successfully synthesized and structurally confirmed by FT-IR, ^1^H NMR, ^13^C NMR, and elemental analysis.
- The **QA** derivatives, particularly *m-*CH₃-QA, demonstrated significant effectiveness as levelling agents, reducing surface roughness by up to **83.24%**.
- The adsorption of **QA** on C-steel surfaces involved a hybrid **physisorption-chemisorption** mechanism, supported by thermodynamic and isotherm analyses (Langmuir, Flory–Huggins, Kinetic, and Dubinin-Radushkevich models).
- The inhibition efficiency (%IE) followed the order: ***m-*CH₃-QA > *o-*Cl-QA > *p*-(CH₃)₂N-QA**, attributed to differences in molecular structure and substituent groups.
- SEM, EDX, AFM, and WCA measurements revealed that **QA** derivatives formed a protective layer on C-steel, enhancing hydrophobicity and reducing corrosion.
- AFM results showed a significant decrease in surface roughness (**Ra**) from 540.2 µm (raw) to 4.67 µm (***m*-CH₃-QA**-treated), corroborating the levelling effect.
- DFT calculations identified reactive sites and electronic properties (HOMO–LUMO energies, Fukui indices, MEP) that explained the superior performance of ***m*-CH₃-QA**.
- The compound exhibited the highest HOMO energy (− 6.34 eV) and smallest energy gap (4.70 eV), indicating strong electron-donating ability and reactivity.
- The findings confirm the efficiency, eco-friendliness, and practical applicability of **QA** derivatives as EP levellers.

Future research could focus on modifying molecular structures to enhance adsorption strength and compatibility with large-scale EP systems.

## Supplementary Information

Supplementary Information.

## Data Availability

The data used and analyzed during the current study are available from the corresponding authors upon reasonable request.
