# Supplementary material for: Theoretical and electrochemical performance of Quinazoline Schiff-base hybrid as levelling agents for C-steel electropolishing in acidic medium
Source: Sci Rep. 2025 Nov 20;15:41109. doi: 10.1038/s41598-025-25001-8 (PMC12635372; doi:10.1038/s41598-025-25001-8)
Supplement: Supplementary file 1 — Supplementary Information. [file 41598_2025_25001_MOESM1_ESM.docx]

**Theoretical and electrochemical performance of Quinazoline Schiff-base hybrid as levelling agents for C-steel electropolishing in acidic medium**

Amira H.E. Moustafa^*^, Hanaa H. Abdel-Rahman, Seleim M. Seleim, Asmaa M. Embaby and Alaa Z. Omar

*Chemistry Department, Faculty of Science, Alexandria University, Alexandria, Egypt*

- **Synthesis of quinazolin-4(3*H*)-one [1]:**

Anthranilic acid (20 g, 0.146 mol) and 2.5 equivalents of formamide (14.5 mL, 0.365 mol) were mixed and irradiated at 300 W for 10 min in a microwave. The reaction mixture was cooled to obtain compound **1 (Cpd 1),** which crystallized from ethanol to give colorless crystals, 18.11 g (85 %) yield; m.p. 225 °C. **IR (KBr)**: 3204 (N-H**),** 3041 (sp^2^ =C-H), 1703 (C=O, cyclic amide), and 1614 (C=N) cm^-1^. **^1^H NMR** (400 MHz: DMSO-*d6*): *δ* 12.28 (Br.s, 1H, NH, D_2_O exchangable), 8.13 (m, 2H, H-2 and H-5), 7.81 (t, *J* = 7.6 Hz, 1H, H-7), 7.67 (d, *J* = 8.1, 1H, H-8) and 7.52 (t, *J* = 7.5 Hz, 1H, H-6) ppm. **^13^C APT NMR** (101 MHz: DMSO-*d6*): *δ* 161.38 (C=O), 148.99 (C), 145.82 (CH), 134.92 (CH), 127.53 (CH), 127.34 (CH), 126.30 (C), and 122.90 (C) ppm [1].

- **Synthesis of ethyl 2-(4-oxoquinozolin-3(4*H*)-yl)acetate [2]:**

A mixture of quinazolin-4(3*H*)-one (1.46 g, 0.01 mol), potassium carbonate (1.8 g, 0.013 mol), and ethyl bromoacetate (1.1 mL, 0.01 mol) in DMF (20 mL) was refluxed for 2 h. After completion of the reaction, the reaction mixture was poured on crushed ice. The crude product was filtered, washed with water, and crystallized from ethanol to form colorless crystals, 1.74 g (75 %) yield; m.p. 59 °C. IR (KBr): 3060 (sp^2^ =C-H), 2994 (sp^3^ =C-H), 1724 (C=O, ester), 1683 (C=O, cyclic amide), and 1613 (C=N) cm^-1^. ^1^H NMR (400 MHz: DMSO-*d6*): *δ* 8.40 (s, 1H, H-2), 8.16 (d, *J* = 7.8 Hz, 1H, H-5), 7.87 (t, *J* = 7.5 Hz, 1H, H-7), 7.72 (d, *J* = 8.1, 1H, H-8), 7.58 (t, *J* = 7.4 Hz, 1H, H-6) ppm, 4.85 (s, 2H, N-CH_2_), 4.18 (q, *J* = 6.9 Hz, 2H, O-CH_2_) and 1.22 (t, *J* = 7.0 Hz, 3H, CH_3_). ^13^C APT NMR (101 MHz: DMSO-*d6*): *δ* 168.45 (C=O, ester), 160.70 (C=O, lactam), 148.35 (CH), 148.27 (C), 135.25 (CH), 127.90 (CH), 127.70 (CH), 126.47 (CH), 121.61 (C), 61.90 (CH_2_), 47.78 (CH_2_) and 14.40 (CH_3_) ppm. 161.38 (C=O), 148.99 (C), 145.82 (CH), 134.92 (CH), 127.53 (CH), 127.34 (CH), 126.30 (C), and 122.90 (C) ppm [2].

- **Synthesis of 2-(4-oxoquinazolin-3(4*H*)-yl)acetohydrazide 1 [2]:**

A solution of ethyl 2-(4-oxoquinozolin-3(4*H*)-yl)acetate (2.32 g, 0.01 mol) and hydrazine hydrate (1.25 mL, 0.025 mol) in ethanol was boiled for 6 h. After completion of the reaction, the solid product formed was filtered and washed with ethanol without needing further purification to obtain colorless crystals, with a 2.07 g (95 %) yield, m.p. 245 °C. IR (KBr): 3295 (N-H**),** 3151 (NH**_2_),** 3053 (sp^2^ =C-H), 2995 (sp^3^ =C-H), 1685 (C=O, cyclic amide), 1660 (C=O, carbohydrazide), and 1607 (C=N) cm^-1^. ^1^H NMR (400 MHz: DMSO-*d6*): 9.46 (Br.s. 1H, NH, D2O exchangeable), 8.32 (s, 1H, H-2), 8.15 (d, *J* = 7.9 Hz, 1H, H-5), 7.86 (t, *J* = 7.4 Hz, 1H, H-7), 7.71 (d, *J* = 8.1, 1H, H-8), 7.57 (t, *J* = 7.4 Hz, 1H, H-6) ppm, 4.63 (s, 2H, N-CH_2_) and 4.33 (Br.s. 2H, NH_2_, D2O exchangeable). ^13^C APT NMR (101 MHz: DMSO-*d6*): *δ* 166.68 (C=O, hydrazide), 160.67 (C=O, lactam), 149.02 (CH), 148.49 (C), 134.92 (CH), 127.66 (CH), 127.56 (CH), 126.44 (CH), 121.95 (C) and 47.33 (CH_2_) ppm. 161.38 (C=O), 148.99 (C), 145.82 (CH), 134.92 (CH), 127.53 (CH), 127.34 (CH), 126.30 (C), 121.95 (C) and 47.33 (CH_2_) ppm [3].


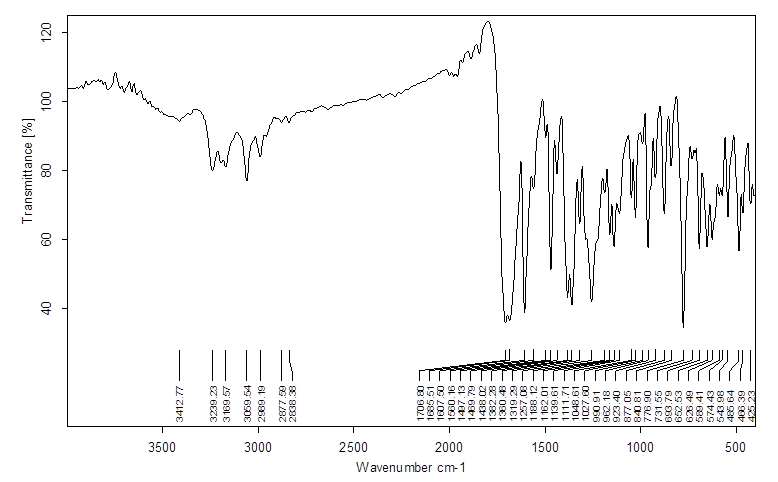


Figure S1. IR (KBr) spectrum of *N*'-(2-Chlorobenzylidene)-2-(4-oxoquinazolin-3(4*H*)-yl)acetohydrazide ***o-Cl*-QA**

Figure S2. ^1^H NMR (400 MHz: DMSO-*d6*) spectrum of *N*'-(2-Chlorobenzylidene)-2-(4-oxoquinazolin-3(4*H*)-yl)acetohydrazide ***o-Cl*-QA**

Figure S3. ^1^H NMR (400 MHz: DMSO-*d6*) spectrum of *N*'-(2-Chlorobenzylidene)-2-(4-oxoquinazolin-3(4*H*)-yl)acetohydrazide ***o-Cl*-QA**

Figure S4. ^13^C NMR (101 MHz: DMSO-*d6*) spectrum of *N*'-(2-Chlorobenzylidene)-2-(4-oxoquinazolin-3(4*H*)-yl)acetohydrazide ***o-Cl*-QA**

Figure S5. ^13^C NMR (101 MHz: DMSO-*d6*) spectrum of *N*'-(2-Chlorobenzylidene)-2-(4-oxoquinazolin-3(4*H*)-yl)acetohydrazide ***o-Cl*-QA**


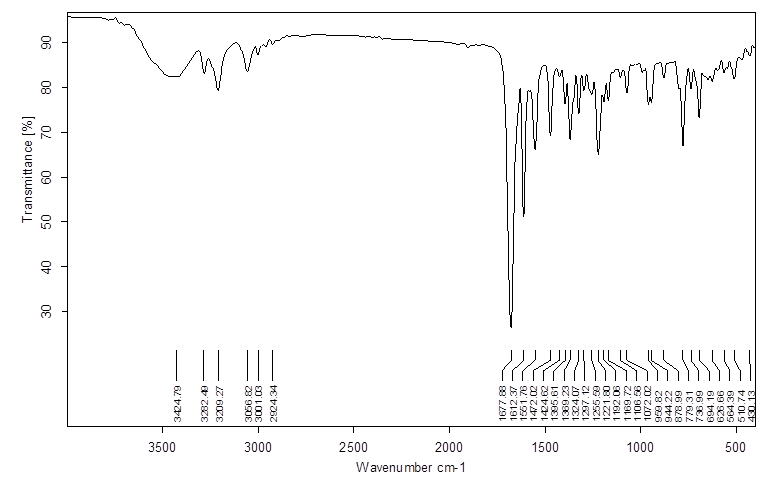


Figure S6. IR (KBr) spectrum of *N*'-(3-Methylbenzylidene)-2-(4-oxoquinazolin-3(4*H*)-yl)acetohydrazide ***m-CH_3_-*QA**

Figure S7. ^1^H NMR (400 MHz: DMSO-*d6*) spectrum of *N*'-(3-Methylbenzylidene)-2-(4-oxoquinazolin-3(4*H*)-yl)acetohydrazide ***m-CH_3_-*QA**

Figure S8. ^1^H NMR (400 MHz: DMSO-*d6*) spectrum of *N*'-(3-Methylbenzylidene)-2-(4-oxoquinazolin-3(4*H*)-yl)acetohydrazide ***m-CH_3_-*QA**

Figure S9. ^13^C NMR (101 MHz: DMSO-*d6*) spectrum of *N*'-(3-Methylbenzylidene)-2-(4-oxoquinazolin-3(4*H*)-yl)acetohydrazide ***m-CH_3_-*QA**

Figure S10. ^13^C NMR (101 MHz: DMSO-*d6*) spectrum of *N*'-(3-Methylbenzylidene)-2-(4-oxoquinazolin-3(4*H*)-yl)acetohydrazide ***m-CH_3_-*QA**


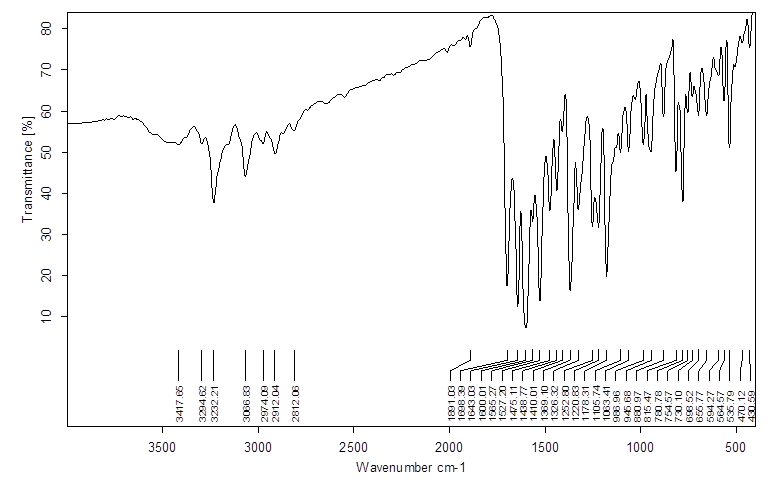


Figure S11. IR (KBr) spectrum of *N*'-(4-(Dimethylamino)benzylidene)-2-(4-oxoquinazolin-3(4*H*)-yl)acetohydrazide ***p-(CH_3_)_2_N*-QA**

Figure S12. ^1^H NMR (400 MHz: DMSO-*d6*) spectrum of *N*'-(4-(Dimethylamino)benzylidene)-2-(4-oxoquinazolin-3(4*H*)-yl)acetohydrazide ***p-(CH_3_)_2_N*-QA**

Figure S13. ^1^H NMR (400 MHz: DMSO-*d6*) spectrum of *N*'-(4-(Dimethylamino)benzylidene)-2-(4-oxoquinazolin-3(4*H*)-yl)acetohydrazide ***p-(CH_3_)_2_N*-QA**

Figure S14. ^13^C NMR (101 MHz: DMSO-*d6*) spectrum of *N*'-(4-(Dimethylamino)benzylidene)-2-(4-oxoquinazolin-3(4*H*)-yl)acetohydrazide ***p-(CH_3_)_2_N*-QA**

Figure S15. ^13^C NMR (101 MHz: DMSO-*d6*) spectrum of *N*'-(4-(Dimethylamino)benzylidene)-2-(4-oxoquinazolin-3(4*H*)-yl)acetohydrazide ***p-(CH_3_)_2_N*-QA**

[1] M.A. El-Atawy, E.A. Hamed, M. Alhadi, A.Z. Omar, Synthesis and Antimicrobial Activity of Some New Substituted Quinoxalines, Molecules, 24 (2019) 4198.

[2] A.Z. Omar, M.A. El-Rahman, S.K. El-Sadany, E.A. Hamed, M.A. El-Atawy, Synthesis of novel bisazo disperse dyes: Spectroscopic characterization, DFT study and dyeing of polyester, Dyes and Pigments, 196 (2021) 109831.

[3] M.A. El-Atawy, A.Z. Omar, M. Hagar, E.M. Shashira, Transalkylidation reaction: Green, catalyst-free synthesis of thiosemicarbazones and solving the NMR conflict between their acyclic structure and intramolecular cycloaddition products, Green Chemistry Letters and Reviews, 12 (2019) 364-376.
